# Random Forest-Based Protein Model Quality Assessment (RFMQA) Using Structural Features and Potential Energy Terms

**DOI:** 10.1371/journal.pone.0106542

**Published:** 2014-09-15

**Authors:** Balachandran Manavalan, Juyong Lee, Jooyoung Lee

**Affiliations:** Center for In Silico Protein Science, School of Computational Sciences, Korea Institute for Advanced Study, Seoul, Korea; CSIR-Institute of Microbial Technology, India

## Abstract

Recently, predicting proteins three-dimensional (3D) structure from its sequence information has made a significant progress due to the advances in computational techniques and the growth of experimental structures. However, selecting good models from a structural model pool is an important and challenging task in protein structure prediction. In this study, we present the first application of random forest based model quality assessment (RFMQA) to rank protein models using its structural features and knowledge-based potential energy terms. The method predicts a relative score of a model by using its secondary structure, solvent accessibility and knowledge-based potential energy terms. We trained and tested the RFMQA method on CASP8 and CASP9 targets using 5-fold cross-validation. The correlation coefficient between the TM-score of the model selected by RFMQA (TM_RF_) and the best server model (TM_best_) is 0.945. We benchmarked our method on recent CASP10 targets by using CASP8 and 9 server models as a training set. The correlation coefficient and average difference between TM_RF_ and TM_best_ over 95 CASP10 targets are 0.984 and 0.0385, respectively. The test results show that our method works better in selecting top models when compared with other top performing methods. RFMQA is available for download from http://lee.kias.re.kr/RFMQA/RFMQA_eval.tar.gz.

## Introduction

The 3D structure of a protein is essential for understanding its function [1]. The success of genome sequencing program resulted in massive amounts of protein sequence data [2]. However, the majority of its 3D structures remain undetermined. Determination of these uncharacterized protein structures by experimental methods such as X-ray crystallography, NMR and electron microscopy is quite difficult and time consuming with high costs. On the other hand, to complement experimental methods, computational methods to predict the 3D (three-dimensional) structure of a protein from its sequence information have been developed. Due to the advances in computing power, it is often possible to generate numerous alternative models for a given protein sequence with little computational burden. However, selecting the best model from the candidate pool remains as a challenging task [3].

Many protein structure prediction methods have been developed and tested in the Critical Assessment of protein Structure Prediction (CASP) experiments [4], [5]. Currently, most of the methods, such as I-TASSER [6], [7], PMS [8] and Rosetta [9] adopt the sampling-and-selection strategy. The first step is to generate a large number of 3D models with a sampling procedure and the second step is to apply model quality assessment programs to identify the most native-like conformation. In many cases, the tools fail to select the best model. Therefore, ranking the predicted structural models correctly is an important problem in structural bioinformatics. To overcome such difficulties, in this study, we devised a new global quality evaluation method by using the random forest machine learning method.

The scoring functions for evaluating the qualities of given 3D models of a protein can be classified into four categories: physics-based potential functions, statistical potential functions, consensus-based functions, and machine-learning-based functions. Physics-based potential functions calculate the energy of a model including its interaction with the solvent according to physical laws [10], [11]. This method is time-consuming and often quite sensitive to small atomic changes. Statistical potential functions evaluate a model based on the statistical information of structural attributes extracted from the database of known protein structures [12]–[17]. However, statistical potential functions only reflect average properties of known protein structures and have limited discriminating power for ranking structural models. Consensus-based functions [18]–[21] perform successfully when most of the models in the pool are similar to the native structure. However, if poor models dominate the model pool, they tend to perform worse than knowledge-based approaches. In addition, consensus-based methods may fail when the consensus between models is low. Machine learning algorithms, such as support vector machine (SVM), neural network (NN) and random forest (RF) evaluate model quality according to learned “rules” [22]–[25]. Various attributes extracted from the sequences and structures of proteins are used as input features, and the model quality is obtained from them. The advantage of machine learning methods is that it considers a large number of attributes simultaneously, and can capture a hidden relationship between them, which is hard to be revealed by statistical potentials.

In this study, we have developed an RF-based Model Quality Assessment (RFMQA) method to estimate the “relative” quality of a set of model protein structures. RFMQA combines statistical potentials as well as the consistency measure between structural features extracted from the 3D coordinates of a protein and predicted values from the protein's primary sequence. Combining several statistical potential terms is a popular strategy that covers various aspects of protein structures and this procedure has been shown to outperform single potential approaches [18], [20], [21], [26]. In RFMQA, we consider three statistical potentials: dDFIRE, Rwplus and GOAP [13]–[16]. In addition, the consistency of secondary structure and solvent accessibility are also used as input features. A relative TM-score [27] is given as the output of the machine and used to rank given models. We show that RFMQA outperforms single-model methods as well as consensus methods in discriminating the best model, and a good correlation exists between the TM-score of the model selected by RFMQA and that of the best model.

## Materials and Methods

### Dataset

In this work, we used the single domain targets of CASP8 (85) and CASP9 (72) as well as individual domains from the multi-domain targets, according to the domain definition of CASP8 (79) and CASP9 (75). The final dataset contains 164 and 147 domains from CASP8 and CASP9, respectively. Both template-based and template-free modeling targets were included. All sever models were downloaded from the CASP website (http://predictioncenter.org/download_area/).

For training of RFMQA, we screened out significantly bad models, for which models are sorted according to their TM-scores [27], and only the top 50% of the models are used. It should be noted that the screening was performed only for the training of our machine. All the benchmarking and testing was done without the screening procedure. In addition, we excluded targets whose average TM-score is less than 0.3. The final dataset contains 229 domains (121 from CASP8 and 108 from CASP9) and 36575 server models.

### Feature extraction

In this study, we used 9 features, 3 from potential energy terms, 4 from secondary structures and 2 from solvent accessibility. These features are as follows:

#### a) Potential energy calculation (3 features)

Three statistical potentials were used as input features: dDFIRE, GOAP, and RWplus. These potential energies evaluate the structural models from different perspectives. dDFIRE is based on the distance dependent pairwise energy term, DFIRE, and the orientation between atoms (polar-polar, polar-nonpolar) involved in the dipole-dipole interaction [13], [14]; GOAP includes DFIRE and additional angle dependent terms [16]; RWplus is a pair-wise distance-dependent atomic statistical potential, which uses an ideal random-walk chain as the reference state [15]. It should be noted that in dDFIRE and GOAP, the identical DFIRE is included.

#### b) Protein secondary structure (4 features)

The consistency between predicted and actual secondary structures of a protein is a good indication of the model quality. For each 3D model, we used DSSP to calculate its secondary structure [28], [29]. We predicted the secondary structure of the target sequence using PSIPRED [30]. The number of secondary structural element (α-helix, β-strand and coil) matches was calculated between the DSSP and PSIPRED. These numbers were converted into % helix, % sheet and % coil by dividing them by its total chain length N_res_ to constitute three features.

For each amino acid residue position 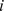, its secondary structure type 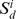 calculated using DSSP is compared with 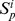 predicted by PSIPRED with the confidence value of 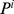. The secondary structure consistency score of a protein 3D model is defined as:

Secondary structure consistency score  = 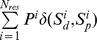


Where 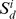, 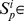 [1], 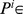 [0, 1] and 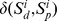 is the Kronecker delta function, which gives 1 if 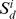 and 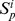 are identical, otherwise 0. The calculated consistency score was used as the fourth feature.

#### c) Solvent accessibility (2 features)

The absolute solvent accessibility (ASA) from the 3D model 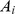 was computed by DSSP [28], [29]. We predicted ASA from the amino acid sequence 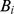 by SANN [31]. These two values were compared and transformed in to a correlation coefficient and cosine value and used as two features. The cosine value is calculated as: 
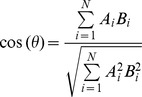
Prior to the training of the Random Forest all feature terms as well as TM-scores were normalized into the range of [0,1] using the following formula:
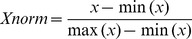



### Random forest

Breiman (2001) proposed Random Forest (RF) [32] as an ensemble technique utilizing hundreds or thousands of independent decision trees to perform classification and regression. RF is a state-of-the-art machine learning technique and has been used for a large number of biological problems [33]–[39]. One important advantage of RF is that it provides the importance information of each input variable, which is suitable for information retrieving from a dataset of high dimension with noise.

The RF algorithm takes an advantage of two ideas, bagging and the random feature selection. For a given training set *D* of size *n*, bagging generates *m* new training sets *D_i_* each of size *n′*, by sampling from *D* uniformly and with replacement, which is called as a bootstrap sample. By using this bootstrap sample, an unpruned regression tree is generated. At each successive node, *m* features are randomly chosen and used to find the best split, which maximizes the information gain measure by Gini impurity [40]. The tree grows until the number of data in the node becomes smaller than the given threshold (cutoff value of 5 is used in this study). Repeating the aforementioned steps to build a large number of regression trees establish the random forest.

The rest of training data, out of bag (OOB) samples, is used to estimate the error rate of the tree as well as the importance of each variable. When a tree is trained, the error of the tree is estimated using the original OOB data. Next, the test feature is randomly permuted among the OOB data and the error of the tree is re-estimated by using the permuted data. The average difference between the two error estimates over all trees in the forest is the raw importance score for the test feature.

For prediction, input features pass through from the root to the end nodes of all trees based on the predetermined splits. The output of each tree is defined as the average objective value in the end nodes and the ensemble average of outputs from all the trees is considered as the final estimate.

### Optimization of the parameters for RF

Several statistical parameters can be tuned to improve the learning in the RF algorithm. In this study, two most influential parameters were the number of trees (n_tree_) used to compute the final average predicted value and the number of variables (m_try_) randomly chosen at each node split. We used the RF regression FORTRAN source code downloaded from the Breiman website (http://www.stat.berkeley.edu/~breiman/RandomForests/reg_home.htm). During each round of cross validation, we optimized the parameters in the following ranges: n_tree_ from 500 to 10000 with the interval of 500 and m_try_ using the values of 1, 2, 3, 4, 5, 6 and 7. Finally, random forest consists of 3000 decision trees and m_try_ = 1 feature was used to obtain the best split at each node providing the optimal performance.

### Benchmark datasets

We used two datasets to test the performance of RFMQA method. The first one constitutes CASP10 server models, which were taken from http://www.predictioncenter.org/download_area/CASP10/server_predictions/. The second one is the full set of I-TASSER decoys [15] downloaded from: http://zhanglab.ccmb.med.umich.edu.

### Evaluation Metrics

We compared the performance of RFMQA with the statistical potential energy terms (dDFIRE, RWplus, GOAP, OPUS and DFIRE). Additionally, we compared our method with top QA methods in CASP10 (GOAP, ProQ2, PMS, ModFOLDclust2, MULTICOM-CONSTRUCT and Pcons) [41]. Among these methods, GOAP, ProQ2 and PMS are single-model methods [8], [16], [42]; ModFOLDclust2, MULTICOM-CONSTRUCT and Pcons are consensus methods [19]–[21], [25], [43]–[46]. The performance was evaluated by four complementary measures: Pearson's correlation coefficient, Spearman's correlation coefficient and the average loss of TM-score between TM_best_ (TM-score of the most native-like structure among decoys) and TM_method_ (TM-score of the model selected by a QA method). The fourth metric is “Z-score”; for each target, TM-score of the model (TM) selected by a QA method was converted into Z-score by dividing (TM – TM_average_) by the standard deviation. Additionally, we computed pairwise comparison between the models selected by TM_RF_ against the models selected by individual methods. Here, TM_RF_ refers to the TM-score selected by RFMQA.

Pearson's correlation coefficient is computed using the following formula:
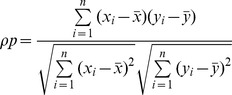


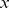
 and 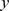 are the TM_method_ and TM_best_, respectively. 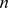 is the total number of targets and 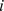 is the target index.

Spearman's correlation is computed using the following equation:
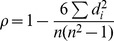
For a given number of targets, the raw scores of 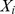 (TM_method_) and 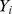 (TM_best_) are converted into ranks of 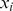 and 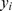. Where 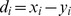, is the difference between the ranks.

## Results and Discussion

In this study, we carried out two model quality assessment experiments using the single domain targets of recent CASP experiments. In the first experiment, a five-fold cross-validation was performed using CASP8 and CASP9 domain targets. The dataset, which contains 229 targets, was randomly divided into 5 groups, and four groups were used for training and the remaining group for testing. This procedure was repeated five times. To obtain the performance of RFMQA from the five-fold cross-validation, the prediction result of each target is calculated using the optimal RF machine generated by using four groups excluding the target. In the second experiment, we evaluated the performance of RFMQA by using the CASP8 and CASP9 as the training dataset and tested the performance against the most recent CASP10 targets.

### Performance of RFMQA during five-fold cross-validation using CASP8 and CASP9 targets

To assess the performance of RFMQA and the other individual statistical potentials, we used four measures: 1) the correlation coefficient (CC_TM_) between the TM-score of the best server model, TM_best_, and the TM-score of the selected model by a QA method, TM_method_, 2) Spearman's correlation coefficient (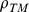) between the TM-score of the best server model, TM_best_, and the TM-score of the selected model by a QA method, TM_method_, 3) the average loss of TM-score, TM_loss_ = TM_best_ – TM_method_, and 4) the average correlation coefficient between predicted ranking and the actual ranking of all targets (CC_rank_) (See Table S1 target details). From Table 1, it is evident that RFMQA outperforms the other statistical potentials in selecting the best model. The average loss of TM-score by RFMQA is 0.055, while the corresponding values of the other statistical potentials are all over 0.06. The better performance of RFMQA demonstrates that combining information from multiple statistical potentials as well as secondary structure and solvent accessibility prediction can give better results than using a single statistical potential. Among the five statistical potential energy terms, dDFIRE outperforms other potentials in selecting the best server model with the TM_loss_ of 0.06.

**Table 1:** Performance of various scoring functions in predicting the quality of the model for five fold validataion.

	RFMQA	dDFIRE	GOAP	DFIRE	RWplus	OPUS	RFMQA (3 features)
CC_TM_	**0.945**	0.919	0.916	0.904	0.912	0.921	0.923
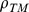	**0.960**	0.952	0.939	0.930	0.932	0.937	0.941
Average TM_loss_	**0.055**	0.060	0.063	0.068	0.063	0.066	0.066
CC_TM_	0.339	0.326	0.333	**0.349**	0.345	0.235	0.271

Note: The first, the second and the third rows respectively represent the correlation coefficient (CC_TM_), Spearman's correlation coefficient (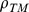) and the average TM-score loss (TM_loss_) between TM_select_ (TM-score of the model selected by QA method) and TM_best_ (TM-score of the best server model). The final row represents the average correlation coefficient between the predicted ranking and the actual ranking (CC_Rank_) over 229 targets. Bold fonts denote the best result.

**Figure 1:**
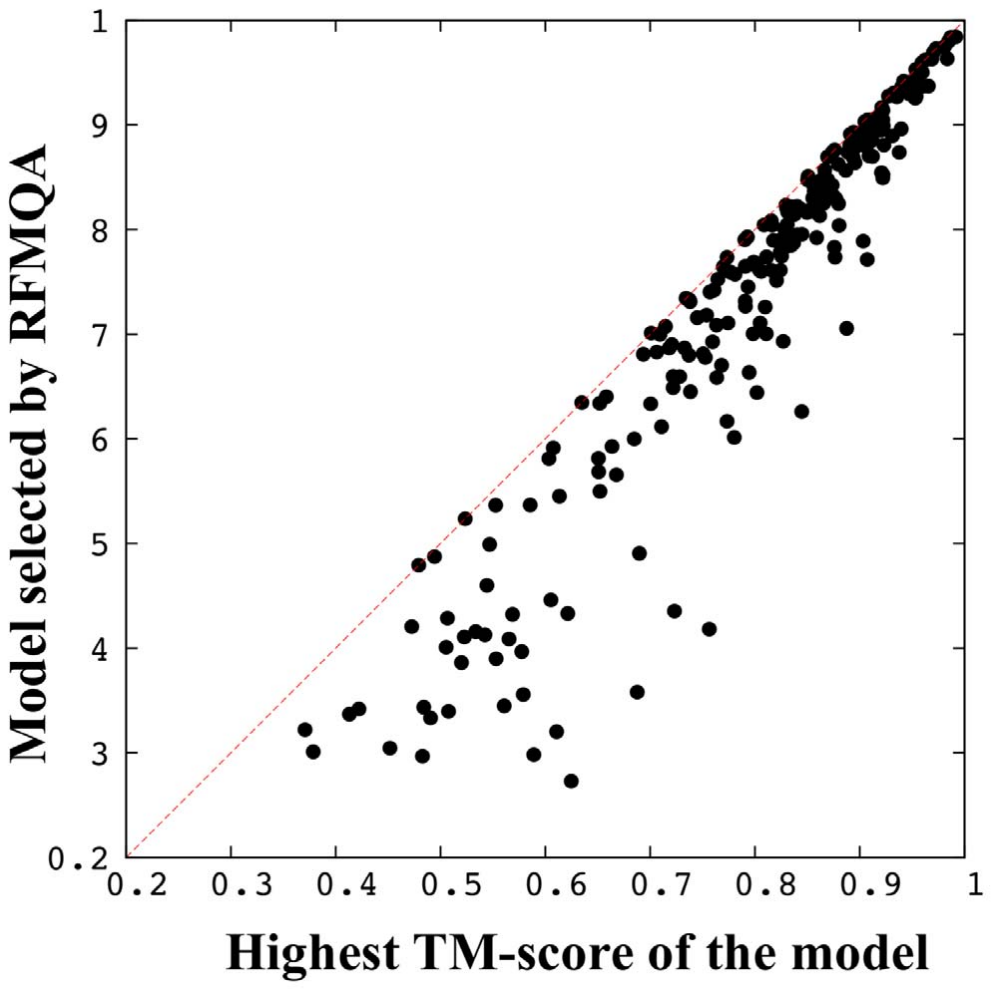
Five-fold cross-validation on CASP8 and CASP9 targets. TM-score of the best server model (TM_best_) versus TM-score of the model selected by RFMQA (TM_RF_) for five-fold validation is shown. Pearson's correlation coefficient and the average TM_loss_ between TM_best_ and TM_RF_ are 0.945 and 0.055, respectively.

A comparison of TM_RF_ and TM_best_ score is illustrated in Figure 1. TM_RF_ shows a good correlation with TM_best_ with CC_TM_ of 0.945 and 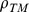 of 0.965. This strong correlation indicates that RFMQA can successfully rank the relative structural qualities of protein 3D models and identify the best model accurately. Moreover, we trained another new RFMQA by using only the statistical potential energy terms as input features. The resulting CC_TM_ and TM_loss_ values are 0.923 and 0.066 respectively. These metrics are slightly worse than the values obtained by RFMQA with 9 features (Table 1), indicating that the combination of various potential energies and consistency between predicted and calculated values from 3D models improves the performance.

**Figure 2:**
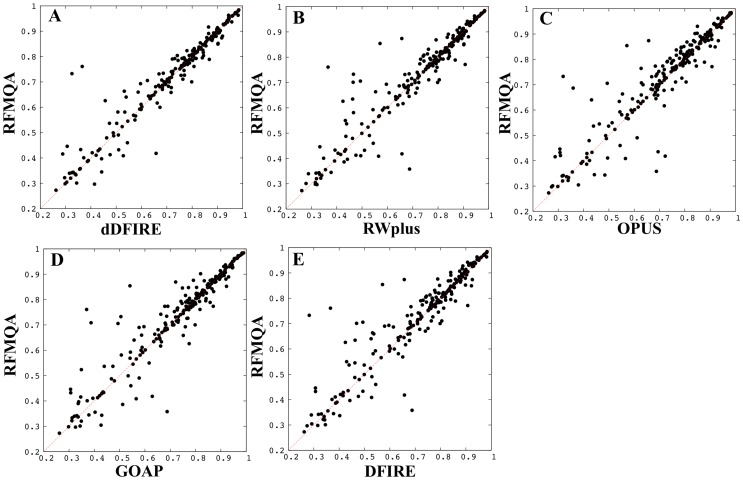
Pairwise comparisons. TM_RF_ against TM-score of the model selected by individual statistical potential (TM_QA_) is shown; (A) dDFIRE versus RFMQA, (B) RWplus versus RFMQA, (C) OPUS versus RFMQA, (D) GOAP versus RFMQA, and (E) DFIRE versus RFMQA.

One of the advantages of random forest method over other machine learning technique is that the importance of input features can be readily obtained during the training. The importance estimation results are shown in Table 2. The results show that the contribution of 9 features is more or less equal. To get more detailed view on the performance of RFMQA, we performed the pairwise comparison of TM_RF_ and TM-score of the model selected by each individual potential energy function (Table 3 and Figure 2). Note that, in Figure 2, the points above the diagonal line correspond to the cases where RFMQA outperforms the other method. The numbers of better and worse predictions by RFMQA compared to each statistical potential, are 105/74 (dDFIRE), 107/77 (RWplus), 130/66 (OPUS), 108/88 (GOAP) and 127/70 (DFIRE). These numbers show that RFMQA model selection is better than the other statistical potential.

**Table 2:** The input features used for RFMQA are listed along with their importance estimates.

Index	Feature	Importance
F1	dDFIRE	26.2
F2	RWplus	30
F3	GOAP	28.7
F4	% of identical α-helix matches between DSSP and PSIPRED	33.3
F5	% of identical β-sheet matches between DSSP and PSIPRED	31.4
F6	% of identical coil matches between DSSP and PSIPRED	27.3
F7	Secondary structure consistency score	22.6
F8	Correlation coefficient of ASA	24.3
F9	Cosine of ASA	25.6

**Table 3:** Pairwise comparisons of RFMQA against individual potential energy terms for five fold cross-validation.

	dDFIRE	GOAP	DFIRE	Rwplus	OPUS
**Gain**	105	108	127	107	130
**Loss**	74	88	70	77	66
**Equal**	50	33	32	45	33

Note: The first row represents the number of models selected by RFMQA that are better than those selected by the potential energy term indicated (Gain). The second row represents the number of models selected by RFMQA worse (Loss) and the third row represents the number of models in tie (Equal).

### Performance on CASP10 targets

To validate the effectiveness of our proposed method, we applied it to the CASP10 targets, where we trained a new RF tree by using the CASP8 and 9 single domain targets as a training set, and the CASP10 targets as a test set (see Table S2). For benchmarking, we utilized 95 targets (QA1; stage2), which were used in the official CASP10 assessment. Prior to the quality assessment, we removed the disordered region in the models predicted by Disopro [47] and subjected those models to quality assessment.

**Figure 3:**
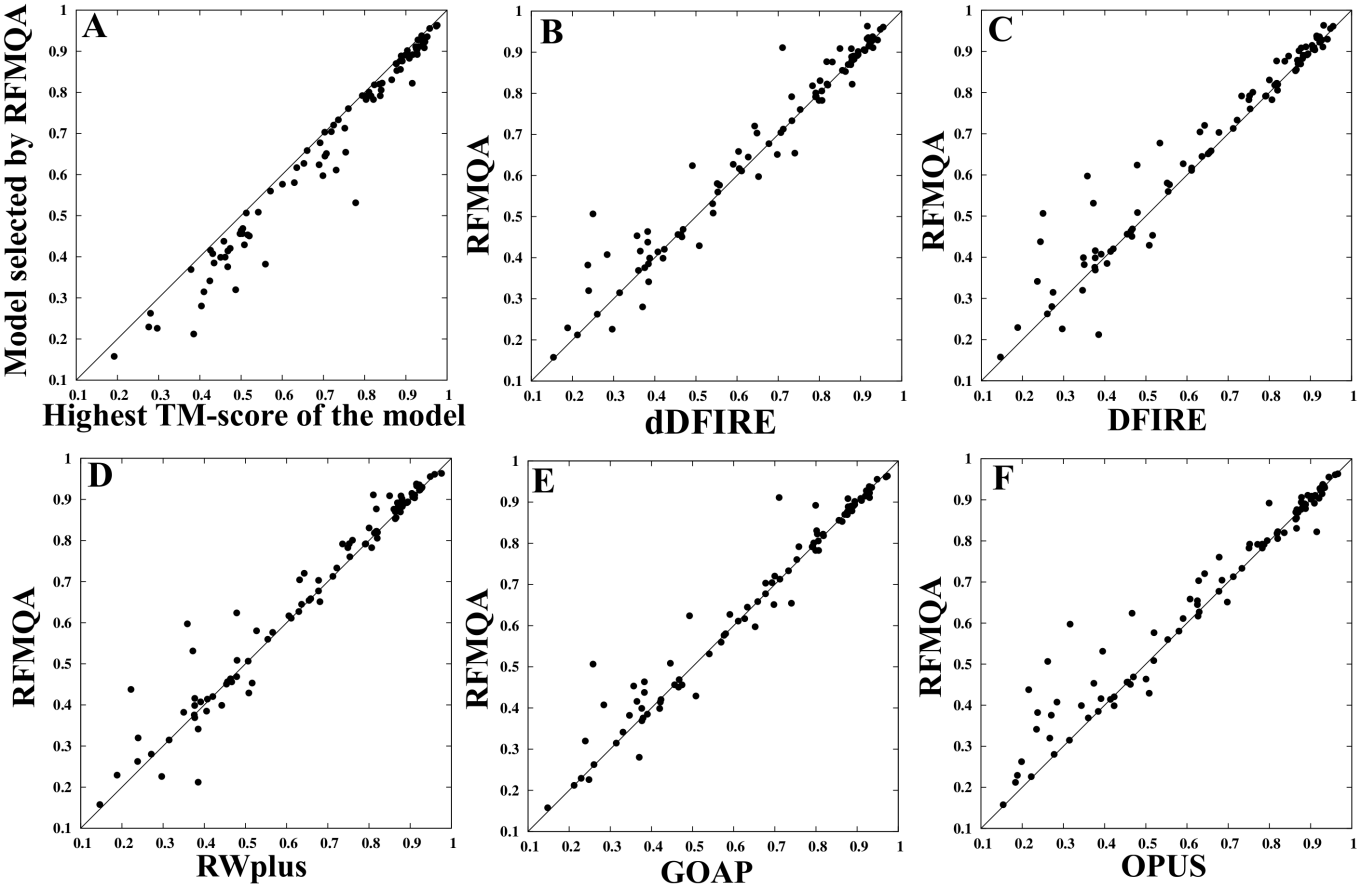
Evaluation of RFMQA on CASP10 targets and its pairwise comparison with other potential energies. (A) TM_RF_ versus TM_best_. Pearson's correlation coefficient and the average TM_loss_ between TM_RF_ and TM_best_ are 0.984 and 0.039, respectively, (B) dDFIRE versus RFMQA, (C) RWplus versus RFMQA, (D) OPUS versus RFMQA, (E) GOAP versus RFMQA, and (F) DFIRE versus RFMQA.

First, we compared the performance of RFMQA with statistical potential energy terms and then with the top QA methods from CASP10. From Table 4, it is clear that RFMQA outperforms the other individual statistical potentials. The average TM_loss_ of RFMQA is 0.038, while that of the best performing statistical potential, GOAP, is 0.049. This difference is more remarkable than the previous 5-fold cross-validation experiment. The pairwise comparison of TM_RF_ with the TM_best_ is illustrated in Figure 3. The CC_TM_ between them is 0.984, while the best performing statistical potential, GOAP, is 0.978. The pairwise comparison of TM_RF_ and TM-score of the model selected by individual energy terms are shown in Figure 3 and Table 4 & 5. The results show that the number of better predictions by RFMQA is larger than those from the other individual statistical potentials.

**Table 4:** Performance of various scoring functions in predicting the quality of the model on CASP10 targets for a blind test.

	RFMQA	dDFIRE	GOAP	DFIRE	RWplus	OPUS
CC_TM_	**0.984**	0.967	0.971	0.956	0.782	0.954
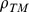	**0.985**	0.964	0.966	0.964	0.960	0.966
Average TM_loss_	**0.038**	0.052	0.048	0.057	0.052	0.061
CC_Rank_	0.395	**0.403**	0.365	0.378	0.357	0.323

Note: The first, the second and the third rows respectively represents the correlation coefficient (CC_TM_), Spearman's correlation coefficient (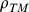) and the average TM-score loss (TM_loss_) between TM_method_ (TM-score of the model selected by a QA method) and TM_best_ (TM-score of the most native-like structure among decoys). The final row represents the average correlation coefficient between the predicted ranking and the actual ranking (CC_Rank_) of 95 CASP10 targets. Bold fonts denote the best result.

**Table 5:** Pairwise comparisons of RFMQA against individual potential energy terms for 95 CASP10 targets.

	dDFIRE	GOAP	DFIRE	Rwplus	OPUS
**Gain**	51	42	59	57	59
**Loss**	28	31	19	23	26
**Equal**	16	22	17	16	10

Note: The first row represents the number of models selected by RFMQA that are better than those selected by the potential energy terms indicated (Gain). The second row represents the number of models selected by RFMQA worse (Loss) and the third row represents the number of models in tie (Equal).

### Comparison of RFMQA with other methods on CASP10 models

CASP10 assessed a variety of model quality evaluation methods including meta methods, clustering methods, energy-based methods and machine learning methods [41], [48], [49]. In this case, we did not try to re-evaluate these methods. Instead, we compared the RFMQA results with top QA (GOAP, ProQ2, PMS, ModFOLDclust2, MULTICOM-CONSTRUCT and Pcons) results from the official CASP10 assessment (http://predictioncenter.org/casp10/qa_analysis.cgi). Among the top QA methods, the ModFOLDclust2 consensus method produced the best results in terms of CC_TM_ (0.979)_,_ average TM_loss_ (0.047) and the TM-score sum of the top model (63.40) (see Table 6). However, RFMQA consistently outperforms ModFOLDclust2 with CC_TM_ of 0.984, average TM_loss_ of 0.038, and the TM-score sum of 64.23. This result shows that our method selects models closer to the native structure than those selected by other methods. In case of CC_Rank,_ ModFOLDclust2 is better than any other methods compared in this study including RFMQA.

**Table 6:** Benchmark of the model quality evaluation on the CASP10 dataset.

Methods	CC_TM_	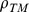	AverageTM_loss_	CC_Rank_	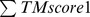	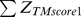
RFMQA	**0.984**	**0.985**	**0.039**	0.396	**64.231**	**111.471**
GOAP	0.979	0.982	0.049	0.488	63.257	78.715
ProQ2	0.978	0.981	0.048	0.404	63.324	84.975
PMS	0.960	0.960	0.058	0.412	80.652	80.652
MULTICOM-CONSTRUCT	0.953	0.962	0.058	0.424	62.347	67.536
ModFOLDclust2	0.979	0.975	0.047	**0.493**	63.408	83.740

Note: The first column represents the method name. The second, the third and the fourth columns respectively represent the correlation coefficient (CC_TM_), Spearman's correlation coefficient (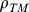) and the average TM-score loss (TM_loss_) between TM_method_ (TM-score of the model selected by a QA method) and TM_best_ (TM-score of the most native-like structure among decoys). The fifth column represents the average correlation coefficient between the predicted ranking and the actual ranking (CC_Rank_). 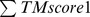 is the sum of the first-ranked models and 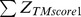 is the sum of Z-score for the first-ranked models. Bold fonts denote the best result.

**Figure 4:**
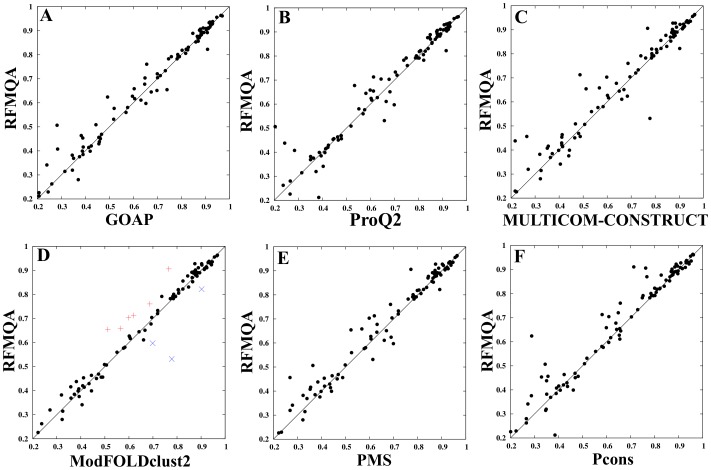
Comparison of RFMQA with top QA methods on CASP10 models. (A) GOAP versus RFMQA, (B) ProQ2 versus RFMQA, (C) MULTICOM-CONSTRUCT versus RFMQA, (D) ModFOLDclust2 versus RFMQA, (E) PMS versus RFMQA, and (F) Pcons versus RFMQA.

To get a detailed view on the performance of RFMQA, we performed the pairwise comparison of TM_RF_ and the TM-score of the model selected by other QA methods (Figure 4 and Table 7). The results show that the number of better predictions by RFMQA is larger than those from the other QA methods. Since, ModFOLDclust2 was the top performer, we compared it with our method in detail as below.

**Table 7:** Pairwise comparisons of RFMQA against top CASP10 methods.

	GOAP	ProQ2	PMS	MULTICOM-CONSTRUCT	ModFOLDclust2	Pcons
**Gain**	51	56	59	59	56	57
**Loss**	31	31	32	34	37	38
**Equal**	13	8	4	2	2	0

Note: The first row represents the number of models selected by RFMQA that are better than those selected by a top QA method (Gain). The second row represents the number of models selected by RFMQA worse (Loss) and the third row represents the number of models in tie (Equal).

The pairwise comparison of RFMQA and ModFOLDclust2 shows that RFMQA gains in 56 cases with an average TM-score gain (TM_RF_-TM_ModFOLDclust2_) of 0.031 and looses in 37 cases with an average TM-score loss of (TM_ModFOLDclust2_ - TM_RF_) 0.025. The benchmarking dataset contain 22 multiple domain proteins (highlighted in magenta in Table S2), where RFMQA is better than ModFOLDclust2 in 16 cases. These results show that RFMQA works well for single domains as well as multiple domains. More specifically, RFMQA selected models better than those by ModFOLDclust2 for the following targets: T0658, T0685, T0698, T0715, T0719, T0743 and T0744 (shown as + in Figure 4D). The average difference in TM-score is 0.109. On the other hand, ModFOLDclust2 performed better for T0700, T0714 and T0742 (shown as x in Figure 4D). Furthermore, we examined the targets with TM-score difference [(TM_RF_ – TM_ModFOLDclust2)_, (TM_ModFOLDclust2_ – TM_RF_)] ≥0.05. Table S3 shows that RFMQA works well in 8/8 cases for the class of alpha+beta proteins; 1/3 case for the class of all-alpha proteins and 1/2 case for the class of all-beta proteins. Overall, our results indicate that RFMQA selects, on average, better models than ModFOLDclust2.

**Figure 5:**
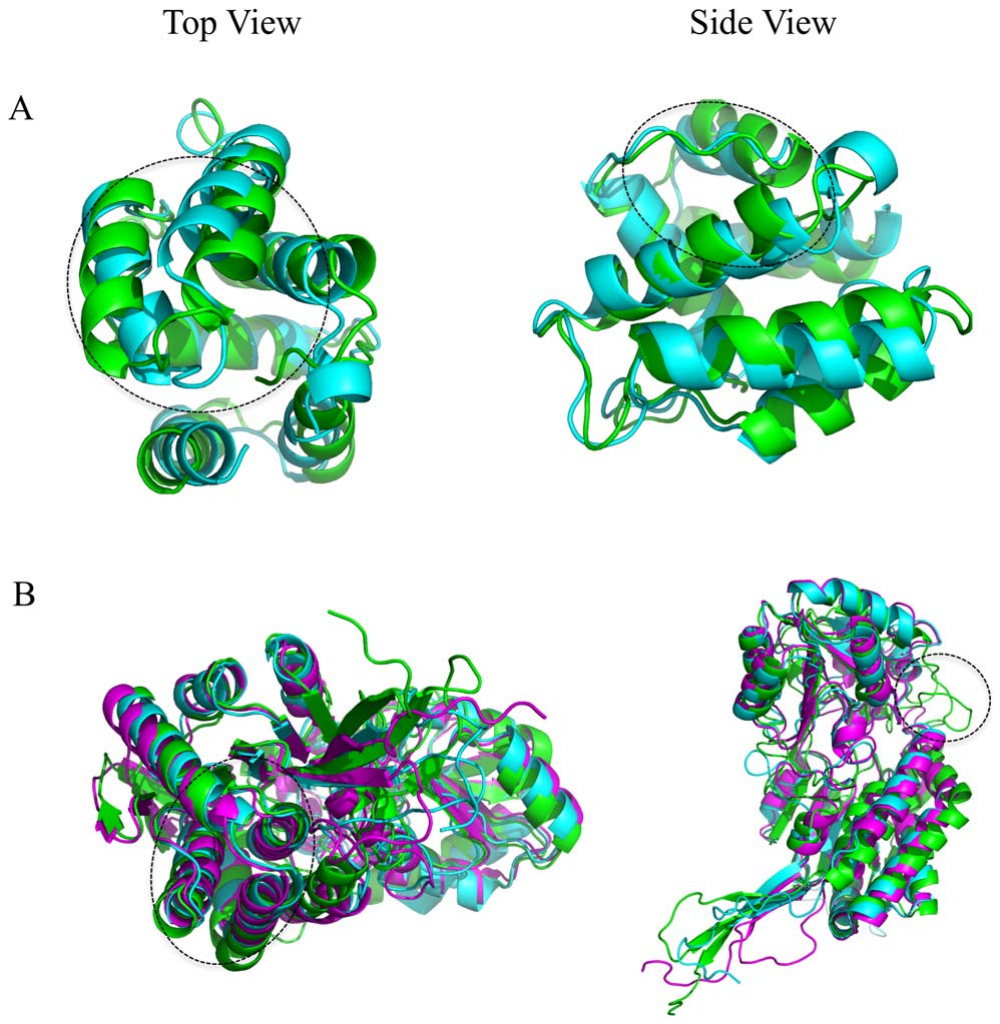
Examples of good predictions by RFMQA are shown for (A) T0698 and (B) T0715. Models selected by RFMQA (magenta) and ModFOLDclust2 (green) are shown as superposed against the TM_best_ model (cyan).

Two examples of better predictions by RFMQA over ModFOLDclust2 are shown in Figure 5. Models selected by RFMQA (magenta) and ModFOLDclust2 (green) are shown as superposed against the TM_best_ model (cyan) for targets T0698 and T0715. Since the RFMQA-selected model is identical to the TM_best_ model in the case of T0698, we compared TM_best_ with ModFOLDclust2. Figure 5A shows that the model selected by ModFOLDclust2 is problematic at the N- and C-terminal helix-helix packing with a slight deviation in the loop region between the helices. Another example is an α+β protein shown in Figure 5B (T0715), where the model selected by ModFOLDclust2 is problematic at the N-terminal region helix-helix packing (see top view). Side view shows that in the middle region (shown inside a circle) it has a long loop instead of extending a helix. On the other hand, the RFMQA model is quite similar to the TM_best_ model with only small deviation in the loop between two helices.

**Figure 6:**
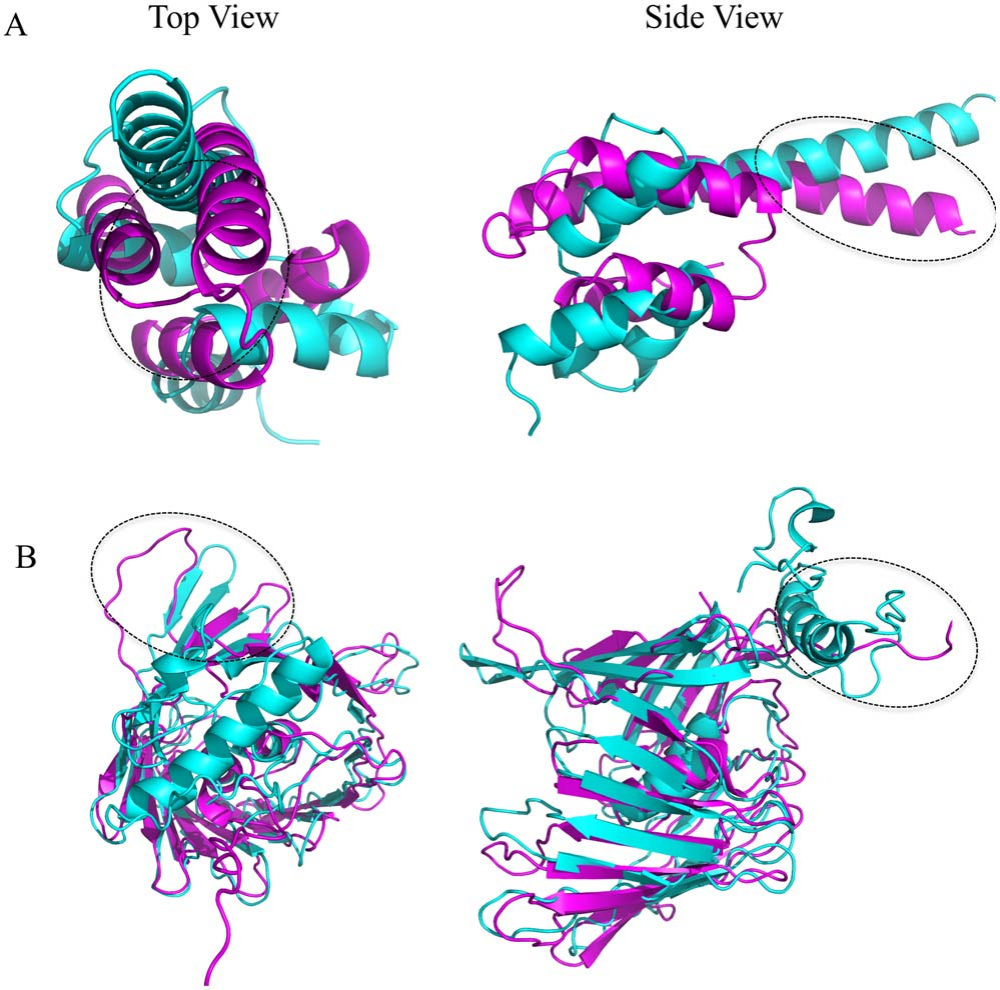
Examples of bad predictions by RFMQA are shown for (A) T0700 and (B) T0742. Models selected by RFMQA (magenta) is shown as superposed against the TM_best_ model (cyan).

Examples of worse predictions by RFMQA are shown for T0700 and T0742 in Figure 6. Since the ModFOLDclust2-selected model is identical to the TM_best_ model in both cases, we compared the TM_best_ model with the RFMQA model. Figure 6A shows that the RFMQA model is problematic in helix-turn-helix packing (top and side views). The lower panel shows that the RFMQA model for T0743 (Figure 6B) is problematic at the N-terminal region, where it has a long loop instead of helix-turn-helix motif (side view; marked in circle). Top view shows that loop connecting the beta-barrel deviates from the TM_best_ model (Figure 6B).

**Figure 7:**
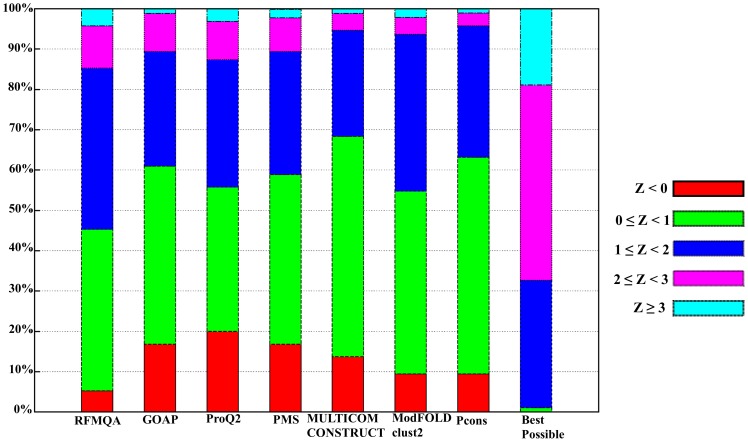
Distribution of Z-score for the model selection on CASP10 targets. Z<0 is colored in red; 0≤Z<1 is colored in green; 1≤Z<2 is colored in blue; 2≤Z<3 is colored in magenta and Z≥3 is colored in cyan.

Furthermore, to analyze the target selection in detail, we calculated Z-score by subtracting the mean quality from the model selected (TM-score) divided by the standard deviation of each target. These Z-scores are not biased by the target difficulty, as the score is normalized by the quality distribution of each target. Hence, it can directly measure the added value of the model quality assessment program relative to a random pick, which would have the value of zero Z-score. Distributions of Z-scores by various methods are shown in Figure 7. The result shows that only 5.3% of RFMQA-selected models are worse than the average (Z<0), while the next best performing Pcons and ModFOLDclust2 have about 9.5% of targets in that range. Conversely, 14.7% of the RFMQA selected model is of high Z-score (Z≥2), while the next best performing ProQ2 has 12.7% in that range. Interestingly, in the figure, all single-model methods select more models with Z≥2 than all consensus methods (ModFOLDclust2, MULTICOM-CONSTRUCT and Pcons). Overall, it shows that RFMQA model selection is consistently better than the other single-model and consensus methods.

### Benchmarking on I-TASSER decoys

In addition to the CASP10 targets, we evaluated the performance of RFMQA on I-TASSER dataset (see Table S4 for detail of targets) to identify the best decoys. The results summarized in Table 8 show that ModFOLDclust2 outperforms the other statistical potential methods such as dDFIRE, RWPlus, OPUS, GOAP and DFIRE in terms of average TM_loss_ (0.095), TM-score sum of the top model (32.588) and Z-score (42.826). However, again, RFMQA is better than ModFOLDclust2 with TM_loss_ of 0.089, TM-score sum of 32.906, and Z-score 44.454. Overall, our result shows that models selected by RFMQA are consistently closer to the native structure than those selected by other QA methods.

**Table 8:** Performance test on the I-TASSER decoy set.

Methods	CC_TM_	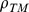	AverageTM_loss_	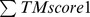	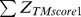
RFMQA	**0.935**	0.913	**0.089**	**32.906**	**44.454**
ModFOLDclust2	0.912	0.908	0.095	32.588	42.826
dDFIRE	0.919	**0.921**	0.099	32.40	39.08
RWplus	0.902	0.920	0.100	32.314	37.069
OPUS	0.883	0.883	0.130	30.652	16.559
GOAP	0.894	0.897	0.115	31.497	28.229

Note: The first column represents the method name. The second, the third and the fourth columns respectively represent the correlation coefficient (CC_TM_), Spearman's correlation coefficient (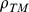) and average TM-score loss (TM_loss_) between TM_method_ (TM-score of the model selected by a QA method) and TM_best_ (TM-score of the most native-like structure among decoys). 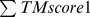 is the sum of the first-ranked models and 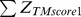 is the sum of Z-score for the first-ranked models. Bold fonts denote the best result.

## Conclusion

In this study, we have developed RFMQA by combining various scoring functions and consistency terms between predicted values and calculated values from 3D models. The current method can predict the relative score of a single model using the potential energy terms and the structural features. The predicted score can be used to rank given 3D protein models and to identify the best model. To evaluate the efficiency of our method, we applied it to recent CASP10 targets. The test results show that RFMQA method is better than other QA methods tested in this study. Its performance generalizes well to different protein targets and structure predictors. Therefore, this new method can be used as a quality assurance component for any protein structure prediction tool.

## Supporting Information

Table S1 **List of CASP8 and CASP9 targets used for 5-fold validation.** Columns from left to right represent the target name, the TM-score of the model selected by RFMQA, dDFIRE, RWplus, OPUS, GOAP and DFIRE. The last column corresponds to the best out of all decoys.
(XLS)

Table S2 **List of CASP10 targets used for benchmarking.** Colums represent the target name, the TM-score of the model selected by RFMQA, GOAP, ProQ2, MULTICOM-CONSTRUCT, ModFOLDclust2, Pcons, PMS, dDFIRE, DFIRE, RWplus and GOAP. The last column corresponds to the best out all decoys. FM targets and Multidomain targets are respectively highlighted in yellow and magenta.
(XLSX)

Table S3 Targets with the TM-score difference greater than 0.05 are listed. The first 11 targets correspond to those with (TM_RF_ – TM_ModFOLDclust2_) ≥0.05 and the last 3 are to those with (TM_ModFOLDclust2_ – TM_RF_) ≥0.05.
(DOCX)

Table S4 **List of I-TASSER decoys used for benchmarking.** Colums represent the target name, the TM-score of the model selected by GOAP, dDFIRE, RWplus, OPUS, DFIRE, RFMQA an ModFOLDclust2. The last column corresponds to the best out all decoys.
(XLSX)
