# Supplementary material for: Random Forest-Based Protein Model Quality Assessment (RFMQA) Using Structural Features and Potential Energy Terms
Source: PLoS One. 2014 Sep 15;9(9):e106542. doi: 10.1371/journal.pone.0106542 (PMC4164442; doi:10.1371/journal.pone.0106542)
Supplement: Table S3 — Targets with the TM-score difference greater than 0.05 are listed. The first 11 targets correspond to those with (TMRF – TMModFOLDclust2) ≥0.05 and the last 3 are to those with (TMModFOLDclust2 – TMRF) ≥0.05. (DOCX) [file pone.0106542.s003.docx]

Table S3.

| **TARGETS** | **Class** | **% of α-helix** | **% of β-sheet** |
| --- | --- | --- | --- |
| T0649 | α+β | 0.196 | 0.255 |
| T0653 | All-β | 0 | 0.261 |
| T0655 | α+β | 0.347 | 0.400 |
| T0673 | All-β | 0 | 0.371 |
| T0685 | α+β | 0.502 | 0.202 |
| T0698 | All-α | 0.597 | 0 |
| T0715 | α+β | 0.407 | 0.207 |
| T0719 | α+β | 0.015 | 0.405 |
| T0743 | α+β | 0.325 | 0.377 |
| T0744 | α+β | 0.338 | 0.172 |
| T0752 | α+β | 0.331 | 0.457 |
| T0666 | All-α | 0.711 | 0 |
| T0700 | All-α | 0.714 | 0 |
| T0742 | All-β | 0.021 | 0.523 |
